# Validation of Fiber-Dominant Expressing Gene Promoters in *Populus trichocarpa*

**DOI:** 10.3390/plants14131948

**Published:** 2025-06-25

**Authors:** Mengjie Guo, Ruxia Wang, Bo Wang, Wenjing Xu, Hui Hou, Hao Cheng, Yun Zhang, Chong Wang, Yuxiang Cheng

**Affiliations:** 1 State Key Laboratory of Tree Genetics and Breeding, Northeast Forestry University, Harbin 150040, China; mengjieguo327@163.com (M.G.); 18632399223@163.com (R.W.); lymyxaa@163.com (B.W.); xuwenjinglucky@163.com (W.X.); houhui@nefu.edu.cn (H.H.); chenghaolucky@126.com (H.C.); z17852423772@163.com (Y.Z.); nefuwangchong@126.com (C.W.); 2 College of Life Sciences, Northeast Forestry University, Harbin 150040, China

**Keywords:** tissue-specific promoter, wood fiber, GUS activity, single-cell RNA sequencing, laser capture microdissection, poplar

## Abstract

Wood is an important raw material for industrial applications. Its fiber-specific genetic modification provides an effective strategy to alter wood characteristics in tree breeding. Here, we performed a cross-analysis of previously reported single-cell RNA sequencing and the AspWood database during wood formation to identify potential xylem fiber-dominant expressing genes in poplar. As a result, 32 candidate genes were obtained, and subsequently, we further examined the expression of these genes in fibers and/or vessels of stem secondary xylem using the laser capture microdissection technique and RT-qPCR. Analysis identified nine candidate genes, including *PtrFLA12-2*, *PtrIRX12*, *PtrFLA12-6*, *PtrMYB52*, *PtrMYB103*, *PtrMAP70*, *PtrLRR-1*, *PtrKIFC2-3*, and *PtrNAC12*. Next, we cloned the promoter regions of the nine candidate genes and created promoter::GUS transgenic poplars. Histochemical GUS staining was used to investigate the tissue expression activities of these gene promoters in transgenic poplars. In one month, transgenic plantlets grown in medium showed intensive GUS staining signals that were visible in the leaves and apical buds, suggesting substantial expression activities of these gene promoters in plantlets predominantly undergoing primary growth. In contrast, for three-month-old transgenic poplars in the greenhouse with predominantly developed secondary stem tissues, the promoters of seven of nine candidate genes, including *PtrMYB103*, *PtrIRX12*, and *PtrMAP70*, showed secondary xylem fiber-dominant GUS signals with considerable spatial specificity. Overall, this study presents xylem fiber-dominant promoters that are well-suited for specifically expressing genes of interest in wood fibers for forest tree breeding.

## 1. Introduction

Wood (i.e., secondary xylem) is one of the most plentiful fixed carbon reservoirs in the terrestrial biosphere, making it an important sustainable feedstock for biofuels and value-added chemicals [1,2,3]. Wood formation undergoes a genetically controlled xylogenesis process of xylem development, which is strongly related to its mechanical qualities, potential as a renewable energy source, and industrial applications. For example, the composition and structure of xylem secondary cell walls (SCW) are important factors influencing wood strength, flexibility, and durability [4]. Fibers, a primary cell type in secondary xylem, play an important role in determining wood characteristics. The thickening and compositional regulation of fiber SCWs are vital for plant structural support as well as industrial uses such as pulp manufacturing and bioenergy [5]. Recent research has identified some critical genes and regulatory networks involved in fiber cell development and SCW biosynthesis in poplar, underlining the possibility of genetically engineering fiber characteristics for industrial use [6,7].

A promoter is a DNA region that is normally found upstream of the coding sequence (CDS). It contains regulatory elements that control gene transcription in response to internal and external stimuli (when, where, how much, and how long). Promoter-reporter systems are commonly employed to examine gene expression and regulation in plants, with reporter genes providing visual and quantitative assessments of promoter activity across developmental stages or circumstances [8,9]. Traditional research has generally used constitutive promoters such as CaMV 35S [10], *OsAct1* [11], and *ZmUbi1* [12] promoters. However, their nonspecific expression typically results in developmental problems, such as reduced yield and delayed flowering [13]. To address these constraints, tissue-specific promoters have received increased attention for their capacity to induce gene expression in specific tissues, reducing transgene leakage, which refers to the unintended spread of transgenes from genetically modified organisms (GMOs) into non-target tissues or organisms, and allowing more precise regulation of target genes. In *Populus*, the xylem-specific promoter *PtrMX3* and the phloem-specific promoter *PtrDP3* accurately control vascular tissue expression [14,15]. Stem-specific *PtrWRKY25* regulates lignin biosynthesis and secondary wall deposition [16], whereas xylem fiber-specific *PtrMYB161* promotes SCW synthesis genes (e.g., CesAs) to boost wood production [17]. Recent advances using fiber-specific promoters (e.g., *proSCFP*) to drive the *GhOR1Del* gene have highlighted their importance by enhancing fiber elongation [18]. Pangenome analysis of *Gossypium barbadense* identified interspecific introgression loci associated with fiber traits, with CRISPR activation of vascular alleles showing targeted improvement potential for plant vascular engineering [19]. Furthermore, Li et al. dissected the shared genetic regulatory basis of fiber quality formation across cotton species via pangenomes for diploid/tetraploid cotton, providing new insights for enhancing fiber quality [20]. Collectively, these advances underscore the need for fiber-dominant promoters in wood formation research, though systematic screening of such promoters remains limited.

Laser capture microdissection (LCM) has dramatically improved the ability to isolate and identify genes from specific cell types. LCM and molecular biology techniques were used to isolate cambial cells in poplar and screen for critical regulatory genes, demonstrating the technique’s usefulness in investigating woody plant development [21,22]. Li et al. employed LCM to isolate xylem fiber cells in poplar and discovered the fiber-specific genes involved in SCW production [23], indicating LCM’s promise for functional genomics research in forest trees. Single-cell RNA sequencing (scRNA-seq) enables precise dissection of cellular heterogeneity and dynamic gene expression profiles in plant tissues, uncovering cell-type-specific regulatory mechanisms and developmental trajectories [24]. Ma et al. constructed a single-cell transcriptional atlas of highly lignified stems, identifying 15 major cell clusters (e.g., xylem, phloem) and revealing gene redundancy during secondary growth in *Populus trichocarpa* [25]. Chen et al. combined scRNA-seq and spatial transcriptomics to analyze vascular development in poplar shoot apices, comparing developmental trajectories between woody (*Populus*) and herbaceous (*Arabidopsis*) plants and identifying conserved and species-specific regulatory networks governing vascular cell fate determination [26]. Du et al. screened for genes with a relatively high expression abundance in the secondary xylem in poplars based on the scRNA-seq technology [27]. These advances have strengthened our knowledge of gene expression patterns of particular cell types and established a solid basis for deciphering transcriptional regulatory networks in plants including trees.

The purpose of this work is to discover the promoters for wood fiber-dominant gene expression in poplar. Based on a cross-analysis of single-cell RNA sequencing (scRNA-seq) and the AspWood database from poplar [23,27,28], 32 genes were identified as potential candidates for dominant expression in xylem fibers. The candidates’ expression in xylem fibers and/or vessels was assessed using the LCM method and RT-qPCR. As a result, nine genes (*PtrFLA12-2*, *PtrIRX12*, *PtrFLA12-6*, *PtrMYB52*, *PtrMYB103*, *PtrMAP70*, *PtrLRR-1*, *PtrKIFC2-3*, and *PtrNAC12*) showed the fiber-dominant expression in secondary xylem. On the basis of cloning promoter regions, creating promoter::GUS vectors, developing transgenic poplars, and histochemical GUS staining, we investigated the activity of these gene promoters in transgenic poplars. Derived from these analyses, this study identified a group of promoters that dominantly drove GUS signals in secondary xylem fibers. In short, this study provides promoters that competently express the gene of interest in wood fibers for the breeding of forest trees.

## 2. Results

### 2.1. Screen of Candidate Genes with Xylem Fiber-Dominant Expression Through scRNA-Seq Data

Single-cell RNA sequencing (scRNA-seq) has emerged as a transformative technology for identifying distinct cell types and transitional states during wood development in woody plants, offering unprecedented insights into the regulatory networks governing cell differentiation. Li et al. combined scRNA-seq with spatial transcriptomics to map 971 genes highly enriched in fibers during secondary growth in poplar, providing a spatial resolution of gene expression patterns (Table S1) [23]. Du et al. elucidated 5408 genes highly enriched in the secondary xylem using scRNA-seq, highlighting the role of these genes in wood formation and cell wall biosynthesis (Table S2) [27].

**Figure 1:**
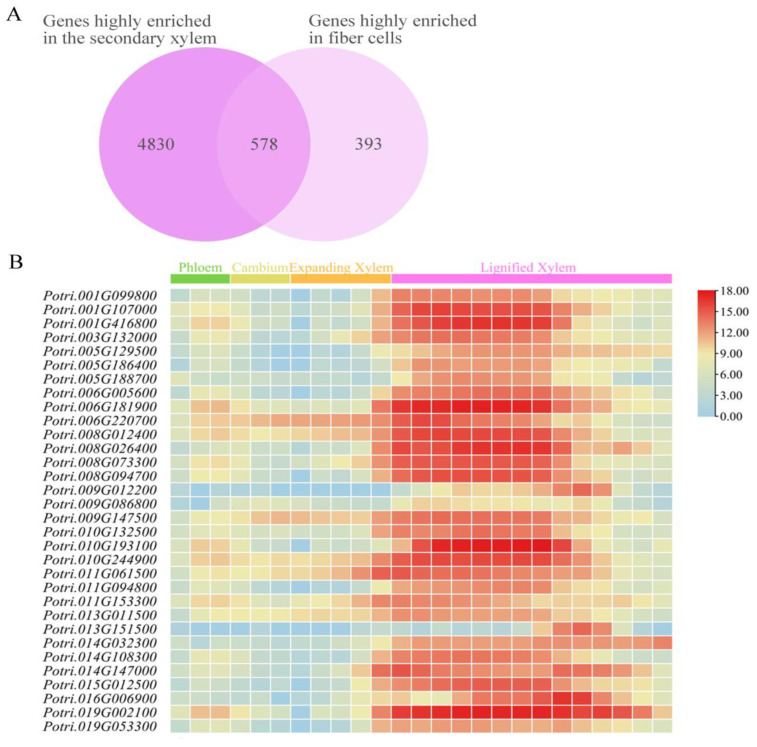
Screening of candidate genes with fiber-dominant expression of poplar xylem (**A**) Venn diagram illustrating the overlap of 578 candidate genes screened from Li and Du’s studies. (**B**) Heatmap of relative expression levels for 32 candidate genes across stem tissues in poplar using Aspwood database [28].

Building on Li and Du’s studies, we initially screened 578 genes highly expressed in xylem fiber cells during secondary growth in *P. trichocarpa* and highlighted in Table S2 (Figure 1A) [23,27]. Subsequently, we analyzed the expression patterns of these genes across different stem tissues according to the AspWood expression database (Table S3) [28]. This analysis refined our selection to 32 candidate genes exhibiting below-average expression in phloem, cambium, and expanding xylem, and above-average expression in lignified xylem (Figure 1B). Furthermore, gene expression profiles (Fragments Per Kilobase of transcript per Million mapped reads (FPKM) values) across tissues were obtained from the Gene Atlas v1 dataset (Figure S1) [29]. These results provide a robust foundation for identifying key regulators of fiber development and secondary xylem formation, offering potential targets for genetic engineering to improve wood quality and biomass yield [4,5].

### 2.2. Expression of Candidate Genes Is Checked in the Fibers and/or Vessels Using LCM Technique and RT-qPCR Analysis

**Figure 2:**
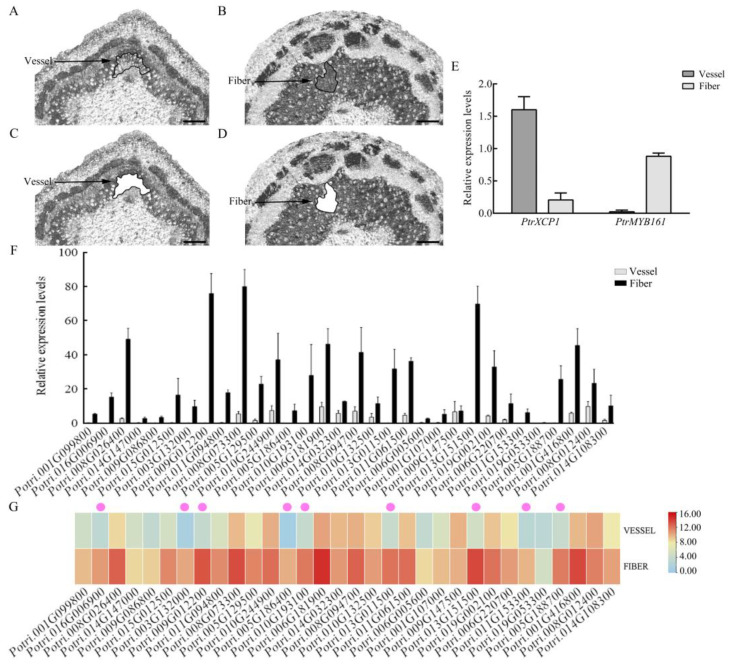
Relative expression levels of 32 screened candidate genes in vessels and fibers. (**A**,**B**) LCM for fiber and vessel cell populations collection. Bars represent 500 μm. (**C**,**D**) The highlighted regions represent cell populations isolated for single-cell RNA analysis. Bars represent 500 μm. (**E**) Expression levels of vessel- and fiber-specific marker genes *PtrXCP1* and *PtrMYB161* in the collected fiber and vessel cell populations. (**F**) Expression profiles of 32 candidate genes in the collected fiber and vessel cell populations, expression level of *PtrActin2* was used as internal reference. (**G**) Heatmap showing the expression patterns of 32 candidate genes in the collected fiber and vessel cell populations, with purple dots indicating the 9 selected genes with high ratio of relative expression levels between fiber and vessel.

To examine the expression profiles of the 32 initially screened genes in fibers and/or vessels, LCM was employed to isolate these specific cell types from the fourth (IN4) and eighth (IN8) internodes of three-month-old wild-type (WT) *P. trichocarpa* (Figure 2A–D). The selections of IN4 and IN8 were in accordance with their distinct anatomical characteristics, with IN4 exhibiting a dense and abundant distribution of vessels and IN8 being enriched in fibers, making them optimal for LCM-based cell type-specific isolation. Actually, the IN8 is a representative secondary stem tissue in poplar as supported by the work of Dharmawardhana et al. [30].

Following LCM, distinct cell populations of IN4-vessel and IN8-fiber were successfully isolated, and RNA was extracted for further analysis. To validate the purity and accuracy of the isolated cell populations, the expression levels of well-characterized cell-type-specific marker genes, *PtrMYB161* (*Potri.007G134500*, fiber-specific) and *PtrXCP1* (*Potri.004G207600*, vessel-specific), were quantified by RT-qPCR [17,31]. The confirmation of high expression levels of the markers in their respective cell types ensured the reliability of the LCM-isolated samples (Figure 2E). Subsequently, the expression levels of the 32 candidate genes were analyzed in LCM-isolated vessels and fibers (Figure 2F,G). Nine candidate genes were selected based on the most pronounced expression divergence between fibers and vessels, showing negligible RNA transcripts in vessels but highly abundant in fibers by comparison.

According to gene annotations in Phytozome 13 and gene descriptions of homologous Arabidopsis genes, the nine candidate genes were defined as *PtrMAP70*, *PtrMYB103*, *PtrFLA12-2*, *PtrMYB52*, *PtrIRX12*, *PtrKIFC2-3*, *PtrFLA12-6*, *PtrNAC12*, and *PtrLRR-1*, respectively (Table 1).

### 2.3. Transcript Levels of These Xylem Fiber-Dominant Expression Genes in Different Tissues

**Figure 3:**
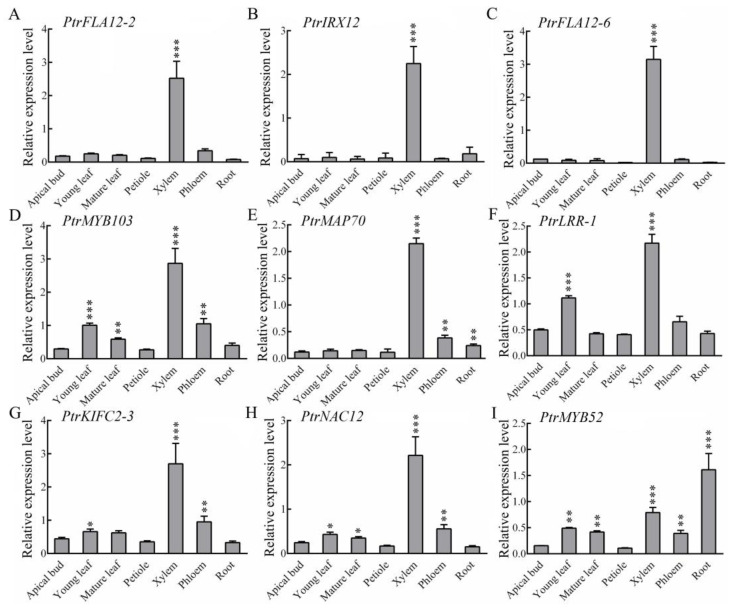
Relative expression levels of nine candidate genes in different tissues. (**A**–**I**) Relative expression levels of *PtrFLA12-2*, *PtrIRX12*, *PtrFLA12-6*, *PtrMYB103*, *PtrMAP70*, *PtrLRR-1*, *PtrKIFC2-3*, *PtrNAC12*, and *PtrMYB52* in apical bud, young leaf, mature leaf, petiole, xylem, phloem, and root. *PtrActin2* expression levels served as the internal reference. Asterisks denote probability levels determined by Student’s *t*-test (*: *p* < 0.05, **: *p* < 0.01, ***: *p* < 0.001).

To further check the tissue-specific expression patterns of nine candidate genes, RT-qPCR was conducted, and the results revealed that mRNAs from the nine candidate genes were highly abundant in developing xylem but exhibited divergent expression levels across other tissues. Specifically, *PtrFLA12-2*, *PtrIRX12*, and *PtrFLA12-6* exhibited xylem-specific high-level expression, indicating their potential roles in xylem development and function (Figure 3A–C). In contrast, *PtrMYB103*, *PtrMAP70*, *PtrLRR-1*, *PtrKIFC2-3*, and *PtrNAC12* in xylem displayed the highest abundance, with moderate expression levels in leaves, phloem, or roots (Figure 3D–H). Unlike the others, *PtrMYB52* showed the highest expression in roots but also maintained considerable expression in xylem (Figure 3I). These findings suggest that nine candidate genes have xylem-dominant expression profiles in *P. trichocarpa*.

### 2.4. Cloning of Gene Promoter Fragments and Production of Promoter::GUS Transgenic Poplars

**Figure 4:**
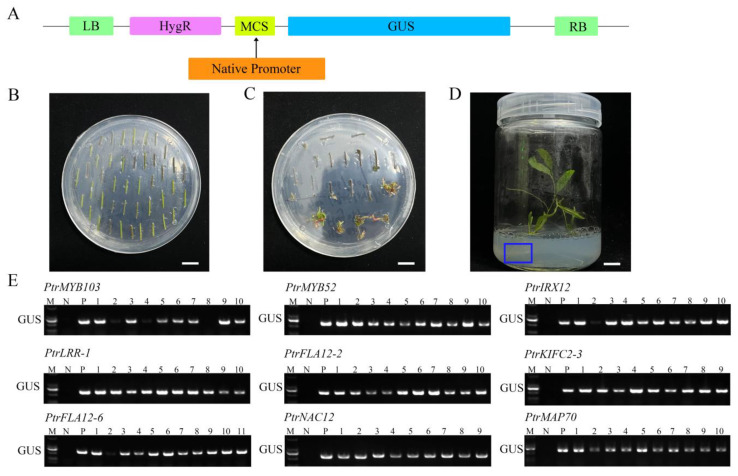
Generation of promoter::GUS transgenic poplar. (**A**) Construction of the promoter::GUS vector. (**B**,**C**) Bud induction culture of promoter::GUS transgenic poplar stem segments on antibiotic-containing selection medium. Bars = 1 cm. (**D**) Rooting culture of transgenic poplar on antibiotic-containing selection medium. Blue boxes indicate normally grown roots. Bars = 1 cm. (**E**) PCR screening of positive promoter::GUS transgenic poplar lines. M, DNA ladder; N, negative controls; P, positive controls; 1–11, transgenic lines.

To test the promoter-driven expression patterns of nine candidate genes, promoter fragments (1.5–2.8 kb upstream of the coding sequences) from these genes were cloned via PCR amplifications (Table S4) and fused to a β-glucuronidase (GUS) reporter gene in pCAMBIA1300 plant expression vector (Figure 4A). These promoter::GUS constructs were transferred into *P. trichocarpa* stem segments via *Agrobacterium tumefaciens*-mediated transformation (Figure 4B). Following antibiotic selection on the culture medium, hygromycin-resistant transformants developing normal shoots and roots were retained (Figure 4C,D). Subsequently, genomic DNA was extracted from these plants, and the transgenes of *PtrMYB103pro::GUS*, *PtrMYB52pro::GUS*, *PtrIRX12pro::GUS*, *PtrLRR-1pro::GUS*, *PtrFLA12-2pro::GUS*, *PtrKIFC2-3pro::GUS*, *PtrFLA12-6pro::GUS*, *PtrNAC12pro::GUS*, and *PtrMAP70pro::GUS* were detected through genomic DNA PCR. The results showed each promoter fragment with the GUS reporter gene in corresponding transgenic plants (Figure 4E). More than six transgenic lines from each transgene were preserved for further studies.

### 2.5. Tissue Expression Activities of Candidate Gene Promoters in Promoter::GUS Transgenic Poplars

**Figure 5:**
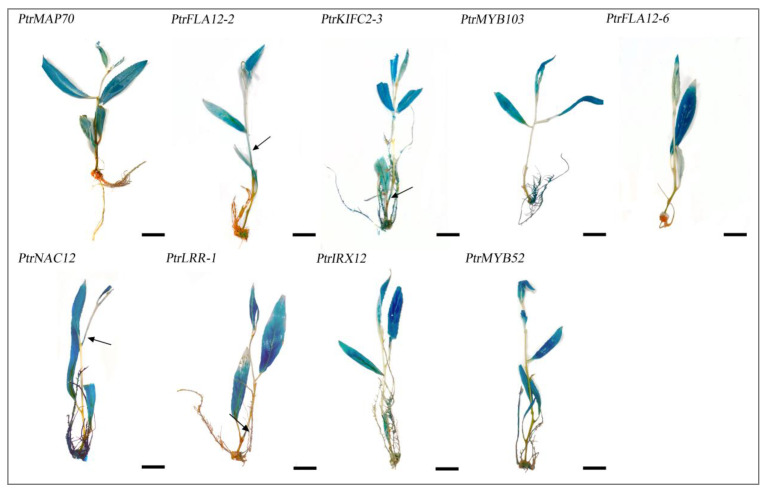
GUS staining in various tissues of the promoter::GUS transgenic poplars. For each gene promoter, six transgenic lines were used for GUS staining analysis, and the experiments were repeated with consistent results. Arrows indicate GUS staining signals in stems. Bars represent 1 cm.

After creating transgenic plants, histochemical staining was used to investigate the tissue expression activities of these gene promoters. In the nine varieties of promoter::GUS transgenic poplars grown for one month in WPM medium, substantial GUS staining signals were seen in the leaves and apical buds during the primary growth phases (Figure 5). Moderate signals were recorded in the stems of transgenic poplars expressing *PtrFLA12-2*, *PtrKIFC2-3*, *PtrNAC12*, and *PtrLRR-1* gene promoters (Figure 5). In addition, we found GUS staining signals in the roots of *PtrKIFC2-3pro::GUS*, *PtrMYB103pro::GUS*, *PtrMYB52pro::GUS*, and *PtrNAC12pro::GUS* transgenic poplars (Figure 5). These findings imply that the nine candidate gene promoters have substantial expression activities in poplars predominantly undergoing initial growth stages.

**Figure 6:**
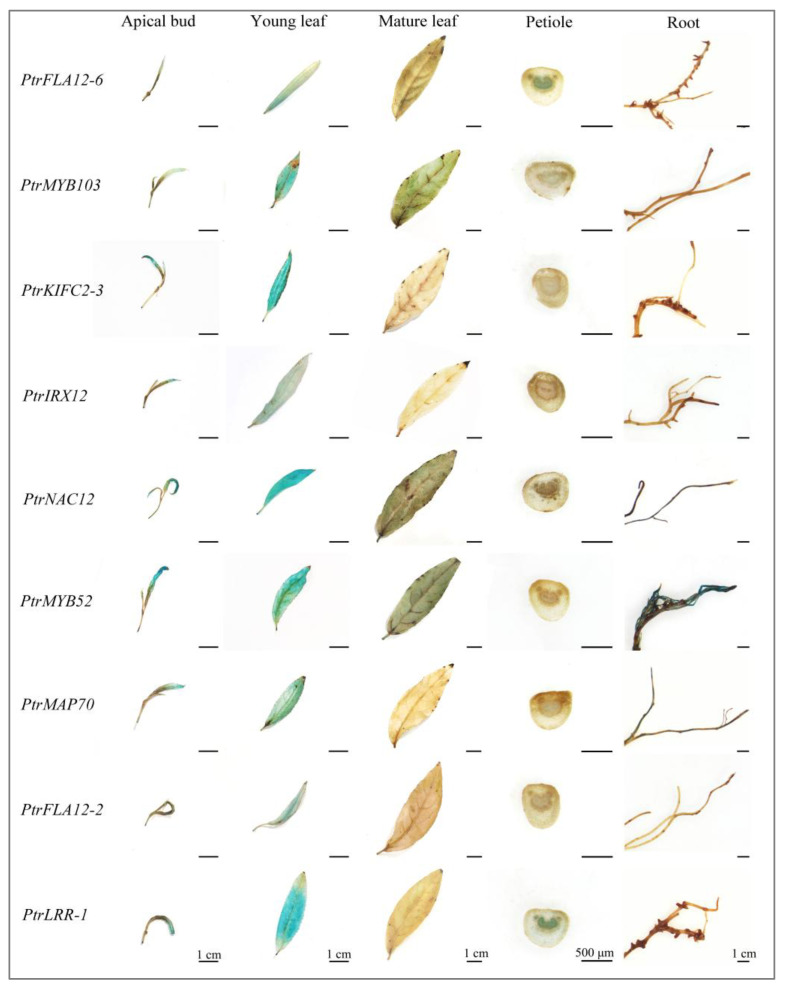
Tissue-specific expression patterns of nine promoter-driven genes in 3-month-old transgenic poplars. GUS activities were analyzed in apical bud, young and mature leaves, petioles, and roots of 3-month-old promoter::GUS transgenic *P. trichocarpa*. For each gene promoter, six transgenic lines were used for GUS staining analysis, and the experiments were repeated with consistent results. Bars = 1 cm or 500 μm.

After three months of growth of transgenic plants on soil, transgenic poplars formed substantial secondary tissues such as mature leaves, developed roots, and lignified stems. We again examined GUS staining activities driven by these gene promoters in promoter::GUS transgenic poplars (Figure 6). GUS signal distribution exhibited striking tissue divergence: high-intensity signals persisted in primary tissues including apical buds and young leaves, while secondary tissues showed promoter-specific activations. GUS staining signals were observed in mature leaves of *PtrMYB103pro::GUS*, *PtrMYB52pro::GUS*, and *PtrNAC12pro::GUS* transgenic poplars. In addition, the petioles of *PtrFLA12-6pro::GUS* and *PtrLRR-1pro::GUS* transgenic poplars and the roots of *PtrMYB52pro::GUS* and *PtrMAP70pro::GUS* transgenic poplars displayed the compartmentalized GUS staining signals.

**Figure 7:**
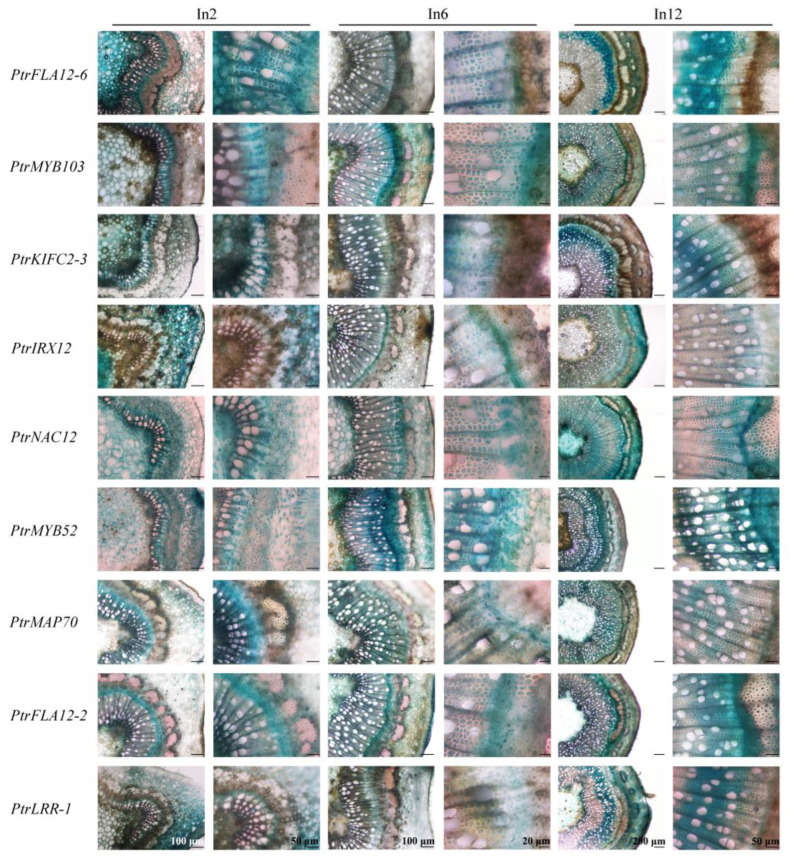
GUS staining in secondary stems of the promoter::GUS transgenic poplars. Cross-sections were prepared from the stained 2nd (dominant primary growth), 6th (exuberant vascular cambia), and 12th (developed secondary growth) stem internodes of 3-month-old promoter::GUS transgenic poplars. For each gene promoter, six transgenic lines were used for GUS staining analysis, and the experiments were repeated with consistent results. The bars of each column of pictures are marked on the bottom row.

To determine the expression profiles of nine candidate genes in xylem fibers, histochemical GUS staining was performed in the 2nd, 6th, and 12th stem internodes of three-month-old promoter::GUS transgenic poplars (Figure 7). The three types of stem internodes underwent distinct developmental stages: dominant primary growth, more exuberant vascular cambia, and developed secondary growth. GUS signals were found across the tissues of the 2nd stem internodes of most transgenic poplars, with the exception of *PtrMAP70pro::GUS* and *PtrFLA12-2pro::GUS* plants, indicating that these promoters’ activities are ubiquitous throughout dominating primary stem growth. In transgenic poplar 6th stem internodes, GUS signals became progressively restricted to developing xylem tissues, and some of promoter::GUS transgenic poplars still exhibited GUS activities in the cambial zone. In the 12th stem internodes, strong and specific blue signals were predominantly localized to xylem cells, exhibiting cell type-specific localization patterns under high-resolution microscopy. To more intuitively demonstrate the GUS activities of nine candidate genes in fiber cells and vessel cells, we performed quantitative analysis (Figure S2) and finally identified seven genes with fiber-dominant expression. Notably, while *PtrFLA12-6pro::GUS* and *PtrFLA12-2pro::GUS* showed GUS activities in both xylem fibers and vessels of the secondary stems, seven kinds of transgenic plants harboring *PtrMYB103pro::GUS*, *PtrIRX12pro::GUS*, *PtrKIFC2-3pro::GUS*, *PtrLRR-1pro::GUS*, *PtrNAC12pro::GUS*, *PtrMYB52pro::GUS*, and *PtrMAP70pro::GUS* exhibited fiber-dominant GUS signals with pronounced spatial specificity. These findings validate seven xylem fiber-predominant promoters in *P. trichocarpa* and underscore their functional relevance in orchestrating fiber-specific transcriptional programs during SCW deposition.

## 3. Discussion

Wood serves as a vital industrial raw material with great potential in the production of both liquid and solid biofuels. The implementation of global renewable energy policies has accelerated the utilization of lignobiomass as an energy source. Given the long generation cycles of tree species, precision breeding through biotechnological approaches is essential to meet escalating demands. Current genetic engineering strategies frequently employ constitutive promoters like CaMV 35S, yet their nonspecific activity often induces detrimental pleiotropic effects that impair plant development. As previously noted, such ubiquitous expression patterns typically manifest in growth abnormalities including yield reduction and flowering delay [13]. A representative example includes *PdGA20ox1*-overexpressing transgenic poplars demonstrating compromised root and leaf development [32]. These observations underscore the critical need to develop tissue-specific promoters for forest trees, enabling targeted genetic modifications and advancing precision breeding programs.

The latest breakthroughs in functional genomics and plant biotechnology have completely transformed the genetic modification strategies of woody plants, enabling enhanced production of traditional forest commodities and next-generation biofuels. With the continuous improvement of transgenic technology, a variety of genetic regulatory factors controlling the biosynthesis pathway of wood have been identified [33,34]. These molecular determinants now constitute the priority targets for regulating the composition and yield optimization of lignocellulosic biomass. The application of tissue-specific promoters provides precise spatiotemporal control for transgenic expression patterns in the context of plant development. Particularly important is the development of vascular tissues including the xylem, cambium, and phloem, which coordinate hydraulic regulation, nutrient transport, and structural integrity maintenance [35]. Notably, the vascular tissue, a physiologically essential system, is also the main site of infection for plant pathogens [36]. As fiber is a primary cell type in vascular tissues, the thickening and composition regulation of its SCW are crucial for plant structural support as well as industrial applications such as pulp manufacturing and bioenergy [5]. Consequently, fiber-dominant promoters provide a complex mechanism to confine transgene activity within specific tissues. This approach minimizes potential pleiotropic effects (though such effects remain speculative and require future validation), while maximizing biological stress resistance and intervention effects in biomass engineering applications.

Building upon existing scRNA-seq datasets and the AspWood expression database [28], this study initially identified 32 candidate genes with elevated expression levels in secondary xylem fibers of *Populus trichocarpa*. To validate these candidates, we performed LCM to isolate fiber and vessel cell populations. Although technical limitations preclude absolute purity of isolated cell types, RT-qPCR quantification of cell type-specific markers (e.g., *PtrMYB161* for fibers, *PtrXCP1* for vessels) confirmed the predominant identity of target cells [17,31]. The integration of scRNA-seq-derived candidates with AspWood’s spatial expression data provides a robust strategy for identifying master regulators of fiber development. Genes exhibiting such spatiotemporal specificity are likely pivotal in orchestrating the transcriptional cascades governing secondary xylem formation, as evidenced by prior functional studies of xylem-specific transcription factors [37,38]. These findings advance our understanding of wood biosynthesis and highlight targets for precision engineering of wood properties (e.g., cellulose content, lignin composition)—critical determinants of industrial biomass quality [4,5].

To elucidate the tissue-specific expression profiles of the nine candidate genes in *Populus trichocarpa*, RT-qPCR was employed to analyze their expression patterns across various tissues (Figure 3). Three genes (*PtrFLA12-2*, *PtrIRX12*, *PtrFLA12-6*) showed xylem-specific expression, aligning with known roles in SCW formation [39]. Conversely, five genes (*PtrMYB103*, *PtrMAP70*, *PtrLRR-1*, *PtrKIFC2-3*, and *PtrNAC12*) displayed higher expression levels in the xylem alongside moderate expression in leaves, phloem, or roots, indicating their potential involvement in both xylem-specific processes and broader physiological functions. This dual expression pattern suggests that these genes may play roles in coordinating developmental and stress responses across multiple tissues, a phenomenon previously documented in other plant species [40,41]. Notably, *PtrMYB52* exhibited the highest expression in roots while maintaining considerable expression in the xylem, implying a dual role in root and xylem development, which echoes the pleiotropic roles of MYB transcription factors in poplar [42]. This finding is also consistent with the evidence suggesting that certain genes involved in vascular development also contribute to root growth and adaptation to environmental stresses [43]. Collectively, these findings highlight the intricate association of the nine candidate genes with xylem development in *Populus trichocarpa*, while also revealing their potential roles in other tissues. The tissue-specific and multi-functional expression patterns of these genes provide valuable insights into the genetic regulation of secondary growth and the integration of developmental processes across different plant organs.

The tissue-specific GUS activity of cloned promoters underscores their utility for precision genetic engineering in modulating wood formation. During secondary growth, promoters such as *PtrMYB103* drove GUS expression exclusively in xylem fibers (Figure 7), consistent with their established roles in regulating SCW biosynthesis through lignin and cellulose deposition [5,17]. As secondary growth progresses, the expression levels of secondary growth-associated genes increase, including the nine candidate genes in this study; their distinct expression patterns between primary and secondary growth stages are likely associated with the developmental process of secondary xylem fibers. The observed GUS activity in primary tissues (Figure 5) seemingly suggests dynamic regulatory mechanisms beyond developmental stages. More reasonably, such pleiotropic expression may reflect hormonal crosstalk, particularly auxin-cytokinin interactions known to modulate cell fate transitions during vascular differentiation [44,45]. For instance, auxin gradients established by PIN transporters in primary tissues could transiently activate SCW-related promoters, as demonstrated in *Arabidopsis* hypocotyls [46]. Notably, the absence of GUS signals in apical buds corroborates the transcriptional suppression of SCW genes in meristematic zones, where stem cell maintenance requires strict inhibition of differentiation programs [47,48]. This spatial restriction ensures proper resource allocation between primary growth and wood formation.

Discrepancies between RT-qPCR data and GUS staining patterns likely arise from multilayered regulatory mechanisms. For example, in this study, the qPCR results of the expression levels of *PtrFLA12-2* and *PtrFLA12-6* in fiber and vessel cells differed from the results presented by final GUS staining, which may be due to post-translational processes, etc. Post-transcriptional controls, such as microRNA-mediated mRNA destabilization (e.g., *miR397* targeting *PtrLAC* genes) [49] or translational buffering [50], may decouple transcript abundance from protein activity.

These xylem fiber-dominant expressing promoters identified provide technical tools for genetically enhancing SCW content in trees, enabling targeted improvements in wood quality and biomass production. However, the trade-off between biomass yield and stress tolerance cautions against unchecked overexpression of SCW-associated genes [51]. Coupling these promoters with stress-inducible regulatory elements could achieve an optimal balance between growth performance and stress resilience [13]. While these promoters have demonstrated fiber specificity in *Populus* species, their potential functional divergence in non-model tree taxa such as *Quercus* requires further investigation [52].

In conclusion, based on previous scRNA-seq studies, the AspWood database, and RT-PCR in fiber and vessel cell populations isolated by LCM, this study identified nine candidate genes. Through the production of promoter::GUS transgenic poplars and GUS staining, we eventually validated seven xylem fiber-dominant promoters in *Populus trichocarpa*. Our fiber-dominantly expressed gene promoters offer tools to engineer trees with enhanced SCW content, the most abundant reservoirs of fixed carbon in the terrestrial biosphere [1]. By bridging molecular insights with industrial applications, these tools hold significant potential for precision breeding to improve wood quality and biomass yield, particularly for bioenergy applications.

## 4. Materials and Methods

### 4.1. Plant Materials and Growth Conditions

Both wild-type *P. trichocarpa* (Nisqually-1) and transgenic plants were planted under controlled greenhouse conditions at Northeast Forestry University (Harbin, China), with a photoperiod of 16 h light/8 h dark and temperatures regulated between 22 and 24 °C. For in vitro propagation, both wild-type and transgenic plants were propagated on Woody Plant Medium (WPM; PhytoTech Lab, Lenexa, KS, USA) plates supplemented with 2.5% (*w*/*v*) sucrose. Transcript levels of genes in different tissues were measured using 4-month-old wild-type trees, including apical bud, young leaf (1st–3rd nodes), mature leaf (6th–8th nodes), petiole, xylem, phloem, and root. All biological samples were immediately flash-frozen in liquid nitrogen and stored at 80 °C until RNA extraction.

### 4.2. Tissue-Specific Expression Pattern Analysis

To investigate the tissue-specific expression profiles of the candidate genes, transcriptomic data were retrieved from the Gene Atlas v1 dataset [29]. Expression levels of the 32 candidate genes were analyzed by extracting FPKM (fragments per kilobase of transcript per million mapped reads) values across seven distinct tissues: bud, young leaf, mature leaf, root tip, root, stem internode, and stem node. The raw FPKM values were log2-transformed to normalize their distribution and subsequently visualized as a clustered heatmap using Tbtools software (v2.310).

### 4.3. LCM

Sections from internodes 4 and 8 of 4-month-old *Populus trichocarpa* grown in a greenhouse were debarked, cut into 500 μm segments, and frozen in liquid nitrogen immediately. Xylem fiber and vessel cell populations were isolated via laser microdissection (LMD7000, Leica, Wetzlar, Germany) under RNAse-free conditions, following established protocols for lignified tissues [53]. Dissected cells were immediately cryopreserved in liquid nitrogen and stored at −80 °C until processing.

### 4.4. Extraction of RNA, and RT-qPCR

Total RNA was isolated from all collected samples using pBIOZOL (Bio-Flux, Beijing, China) in accordance with the manufacturer’s instructions. All isolated RNA samples were assessed for purity by UV spectrophotometry (NanoDrop OneC, Thermo Fisher, Waltham, MA, USA), requiring A260/A280 ≥ 2.0 and A260/A230 ≥ 1.9. Then, the qualified RNA samples were used to synthesize the cDNAs by reverse transcription using the PrimeScript RT Reagent Kit with gDNA Eraser (TaKaRa, Dalian, China). RT-qPCR analysis was conducted using the qTOWER3G real-time PCR system (Analytik Jena, Jena, Germany) with SYBR^®^ Green I master mix (LABLEAD, Beijing, China). *PtrActin2* served as the endogenous control, and relative gene expression was quantified through the 2^−ΔΔCT^ method [54]. Each experimental condition included three independent biological replicates, with triplicate technical repetitions per replicate. Primer sequences are detailed in Supplementary Table S5.

### 4.5. Vector Construction

The genomic DNA was extracted from the leaves of 3-month-old WT using a plant genomic DNA extraction kit (Bioteke, Beijing, China). With genomic DNA as templates, promoter fragments of all genes were amplified by PCR and inserted into the vector pCAMBIA1300-GUS (Invitrogen, Carlsbad, CA, USA) using an In-Fusion^®^ HD Cloning Kit (Takara, CA, USA) in accordance with the user manual. Promoter regions (1.5–2.8 kb upstream of coding sequences) were selected based on both the coverage of known cis-regulatory elements from homologous genes in *Populus trichocarpa* (PlantPAN4.0 database) and the exclusion of adjacent gene coding regions (via genome annotation). All target vectors were transformed into *Agrobacterium tumefaciens* strain GV3101 for future transformation. All primers are listed in Table S5.

### 4.6. Genetic Transformation

For *Populus trichocarpa* transformation, *Agrobacterium tumefaciens* strains harboring promoter::GUS constructs were cultured following established protocols [55]. Wild-type stem internodes (1 cm length) were agroinfiltrated for 20 min with gentle agitation (30 rpm) in transformation medium, then co-cultivated on WPM supplemented with 2.5% sucrose under dark conditions for 48 h. Post-co-cultivation, explants were transferred to shoot induction medium (WPM + 2.5% sucrose + 10 μg·mL^−1^ hygromycin) under a 16 h photoperiod to select hygromycin-resistant adventitious shoots. Putative transgenic shoots were subsequently transferred to root induction medium (WPM + 2.5% sucrose + 5 μg·mL^−1^ hygromycin) to obtain rooted transgenic plantlets. All procedures were performed with reference to a previous study [56].

### 4.7. Identification of the Transgenic Plants

Genomic DNA was extracted from putative transgenic poplars using a plant genomic DNA extraction kit (Bioteke, Beijing, China). Transgenic poplars were identified with PCR amplification using primers specific to the *GUS* reporter gene. To ensure the accuracy of the PCR verification, we performed PCR using both the negative control (distilled water) and the positive control (vector plasmid) as templates, too. The sequences of primers used for vector construction and transgenic plant identification are provided in Table S2.

### 4.8. GUS Staining

Histochemical GUS assays were performed on 1-month-old (primary growth) and 3-month-old (secondary growth) promoter::GUS transgenic poplar lines grown under greenhouse conditions. Six independent transgenic lines were analyzed to ensure biological reproducibility, with wild-type plants serving as negative controls. Tissues were vacuum-infiltrated in GUS staining solution containing 2 mM X-Gluc (GoldBio), 100 mM sodium phosphate buffer (pH 7.0), 0.1% Triton X-100, and 0.5 mM potassium ferrocyanide/ferricyanide, followed by overnight incubation at 37 °C. After the GUS signal was generated, chlorophyll was removed via sequential 75% (*v*/*v*) ethanol washes until background parenchyma showed no detectable blue pigment. Imaging was conducted using two modalities: the hand cross-sections of poplar leaf veins and the petioles were taken with a model SZX7 stereomicroscope (Olympus, Tokyo, Japan), and other tissues were taken by a BX43 laboratory microscope (Olympus, Tokyo, Japan).

Quantitative comparison of GUS staining intensity between fiber and vessel regions was performed using ImageJ (v1.8.0). Mean gray values (8-bit scale) were measured in both tissue types within the same stem cross-sections, with paired background subtraction using immediately adjacent non-stained parenchyma and normalization to wild-type parenchyma controls. Five independent sections per transgenic line were analyzed across three biological replicates under standardized imaging conditions.

### 4.9. Statistical Analysis

Statistical analyses were performed using SPSS 24.0. The values represent means ± standard deviation (SD) of three biological replicates. Statistical significance between groups was determined by Student’s *t*-test, and asterisks denote probability levels (*: *p* < 0.05, **: *p* < 0.01, ***: *p* < 0.001).

## Figures and Tables

**Table 1:** Nine candidate genes and their definitions.

Gene ID	Define
*Potri.016G006900*	*PtrMAP70*
*Potri.003G132000*	*PtrMYB103*
*Potri.009G012200*	*PtrFLA12-2*
*Potri.005G186400*	*PtrMYB52*
*Potri.010G193100*	*PtrIRX12*
*Potri.013G011500*	*PtrKIFC2-3*
*Potri.013G151500*	*PtrFLA12-6*
*Potri.011G153300*	*PtrNAC12*
*Potri.005G188700*	*PtrLRR-1*

## Data Availability

All experimental data are provided in the main text and/or Supplementary Materials.

## Supplementary Materials

The following supporting information can be downloaded at: https://www.mdpi.com/article/10.3390/plants14131948/s1, Figure S1: Heatmap showing the expression patterns of 32 candidate genes in different tissues; Figure S2: Quantitative ImageJ analysis of GUS staining intensity for nine candidate genes in fibers and vessels; Table S1: High enriched genes in xylem fiber subgroup in *populus*; Table S2: Genes highly enriched in the secondary xylem in poplar; Table S3: Relative expression levels of candidate genes derived from Aspwood database in stem tissues; Table S4: Sequence and length of the promoter fragments of screened nine candidate fiber-dominant expression genes; Table S5: Primers for the study.
