# Supplementary figures and images for: Validation of Fiber-Dominant Expressing Gene Promoters in Populus trichocarpa
Source: Plants (Basel). 2025 Jun 25;14(13):1948. doi: 10.3390/plants14131948 (PMC12252207; doi:10.3390/plants14131948)

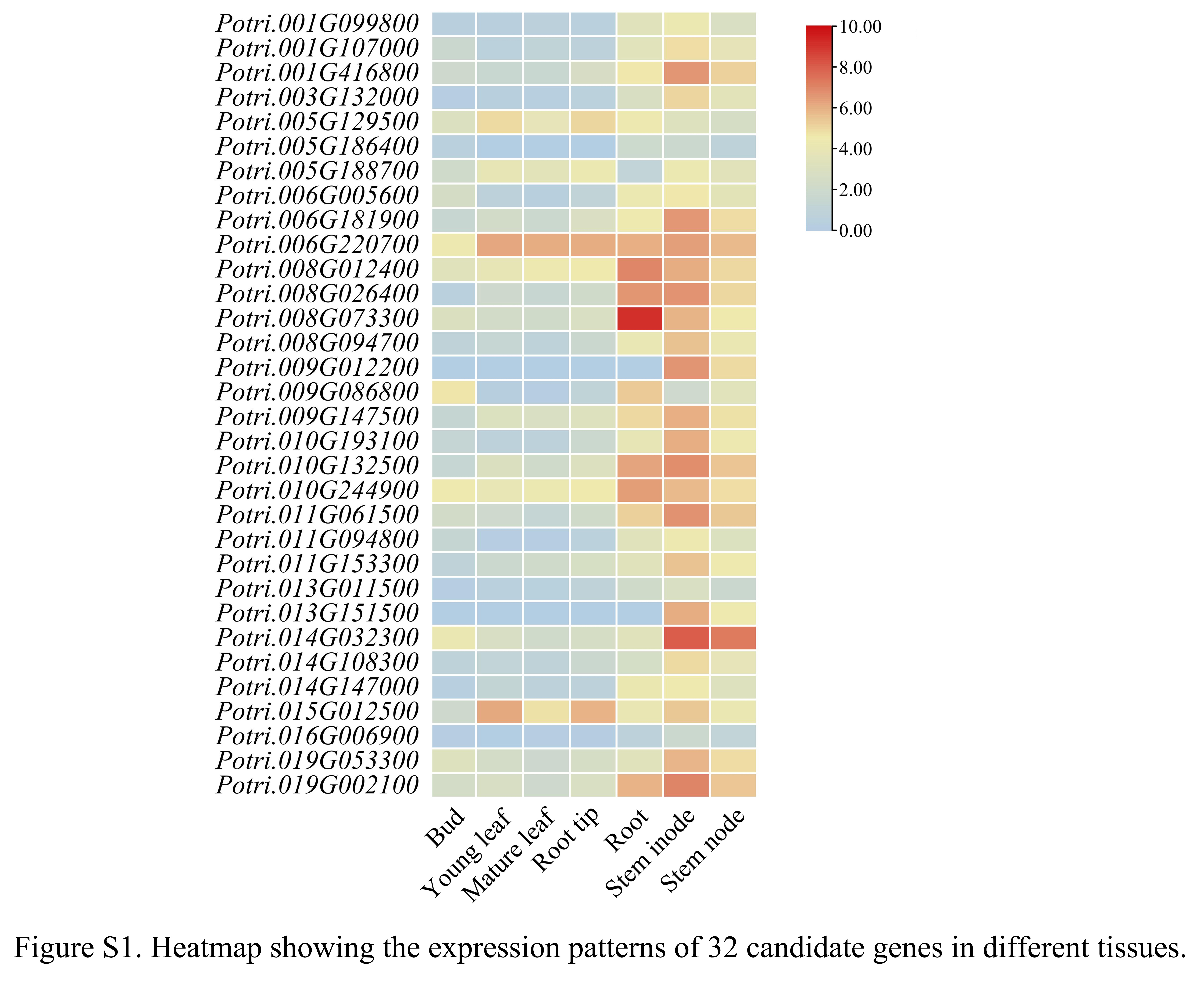

Supplement: Supplementary file 1 [file plants-14-01948-s001.zip › Figure S1.Heatmap showing the expression patterns of 32 candidate genes in different tissues.jpg]
